# Multi-timescale hybrid components of the functional brain connectome: A bimodal EEG-fMRI decomposition

**DOI:** 10.1162/netn_a_00135

**Published:** 2020-07-01

**Authors:** Jonathan Wirsich, Enrico Amico, Anne-Lise Giraud, Joaquín Goñi, Sepideh Sadaghiani

**Affiliations:** Beckman Institute, University of Illinois at Urbana-Champaign, Urbana, IL, USA; EEG and Epilepsy Unit, University Hospitals and Faculty of Medicine of Geneva, Geneva, Switzerland; School of Industrial Engineering, Purdue University, West Lafayette, IN, USA; Purdue Institute for Integrative Neuroscience, Purdue University, West Lafayette, IN, USA; Department of Neuroscience, University of Geneva, Geneva, Switzerland; School of Industrial Engineering, Purdue University, West Lafayette, IN, USA; Purdue Institute for Integrative Neuroscience, Purdue University, West Lafayette, IN, USA; Weldon School of Biomedical Engineering, Purdue University, West Lafayette, IN, USA; Beckman Institute, University of Illinois at Urbana-Champaign, Urbana, IL, USA; Psychology Department, University of Illinois at Urbana-Champaign, Urbana, IL, USA

**Keywords:** Brain connectivity, Human connectome, Concurrent EEG-fMRI, ICA

## Abstract

Concurrent electroencephalography (EEG) and functional magnetic resonance imaging (fMRI) bridge brain connectivity across timescales. During concurrent EEG-fMRI resting-state recordings, whole-brain functional connectivity (FC) strength is spatially correlated across modalities. However, cross-modal investigations have commonly remained correlational, and joint analysis of EEG-fMRI connectivity is largely unexplored. Here we investigated if there exist (spatially) independent FC networks linked between modalities. We applied the recently proposed hybrid connectivity independent component analysis (connICA) framework to two concurrent EEG-fMRI resting-state datasets (total 40 subjects). Two robust components were found across both datasets. The first component has a uniformly distributed EEG frequency fingerprint linked mainly to intrinsic connectivity networks (ICNs) in both modalities. Conversely, the second component is sensitive to different EEG frequencies and is primarily linked to intra-ICN connectivity in fMRI but to inter-ICN connectivity in EEG. The first hybrid component suggests that connectivity dynamics within well-known ICNs span timescales, from millisecond range in all canonical frequencies of FC_EEG_ to second range of FC_fMRI_. Conversely, the second component additionally exposes linked but spatially divergent neuronal processing at the two timescales. This work reveals the existence of joint spatially independent components, suggesting that parts of resting-state connectivity are co-expressed in a linked manner across EEG and fMRI over individuals.

## Author Summary

Functional connectivity is governed by a whole-brain organization measurable over multiple timescales by functional magnetic resonance imaging (fMRI) and electroencephalography (EEG). The relationship across the whole-brain organization captured at the different timescales of EEG and fMRI is largely unknown. Using concurrent EEG-fMRI, we identified spatially independent components consisting of brain connectivity patterns that co-occur in EEG and fMRI over subjects. We observed a component with similar connectivity organization across EEG and fMRI as well as a component with divergent connectivity. The former component governed all EEG frequencies while the latter was modulated by frequency. These findings show that part of functional connectivity organizes in a common spatial layout over several timescales, while a spatially independent part is modulated by frequency-specific information.

## INTRODUCTION

The advances in neuroimaging in the last decades have brought new important insights on brain functioning by using both electroencephalography (EEG) data and functional magnetic resonance imaging (fMRI) data extracted from human brain. EEG provides a direct measure of neuronal activity with high temporal resolution but is limited by the spatial coverage of electrodes on the scalp and by volume conductance. Conversely, fMRI provides a high-resolution estimate of brain function with whole-brain coverage, however, with limited temporal reliability restricted by the slow filter of the hemodynamic response (Logothetis et al., 2001) and the time needed to acquire a whole-brain image (typically in the range of 1–3 seconds).

Functional connectivity (FC), measured as statistical dependence of neural activity across distant brain areas, is considered crucial for brain function and cognition. In large-scale brain network models of FC, nodes correspond to gray matter regions (based on brain atlases or parcellations) while links or edges correspond to connections between the nodes. The connectivity between brain regions in fMRI is usually computed as the pairwise Pearson’s correlation between brain regions’ infraslow (<0.1 Hz) time series (Biswal et al., 1995). On a whole-brain level this graph is either called a FC matrix (Achard et al., 2006) or functional connectome (FC_fMRI_) (Sporns, 2011). When looking at the whole-brain functional connectivity of the human brain, a stable pattern of interconnected intrinsic connectivity networks (ICNs) arise that closely resemble co-activation patterns observed during cognitive tasks (Damoiseaux et al., 2006; Fox et al., 2005; Yeo et al., 2011).

In electrophysiological recordings, stable activation patterns arise forming so-called microstates (Koukkou-Lehmann et al., 1980; Lehmann et al., 1987) reorganizing on a fast timescale (∼100 ms). It has been shown that these fast electrophysiological patterns are linked to fMRI ICNs (Britz et al., 2010; Musso et al., 2010). Beyond such activation patterns, to derive electrophysiological *connectivity* in MEG or EEG (FC_M/EEG_), the statistical dependencies between brain regions’ M/EEG activity can be measured by phase coupling, for example, by using the pairwise imaginary part of the coherency (Wirsich et al., 2017) or by amplitude coupling using Pearson’s correlation of the Hilbert envelope (Brookes et al., 2011; Deligianni et al., 2014). These pairwise FC_M/EEG_ measures are estimated for source-reconstructed M/EEG time series at a specific frequency band. In other words, the rich oscillatory repertoire of electrophysiological activity gives rise to multiple frequency-specific connectomes in various canonical frequency bands. Phase- and amplitude-based connectivity measures are reliably related to each other (Colclough et al., 2016), and it has been shown that both organize into ICNs (de Pasquale et al., 2010; de Pasquale et al., 2012) spatially resembling fMRI ICNs (Brookes et al., 2011). Recent concurrent EEG-fMRI studies have directly shown that whole-brain EEG connectivity in all oscillatory frequency bands is spatially related to fMRI connectivity (Deligianni et al., 2014; Wirsich et al., 2017).

Besides this partial connectivity overlap across modalities, brain responses measured from fMRI and electrophysiology show discrepancies not only arising from different signal-to-noise levels, but also because they capture different aspects of brain activity at different timescales (Furey et al., 2006). Those discrepancies can also be observed when comparing multimodal connectivity. Indeed, when compared directly, it was recently shown that modality-specific differences in localized pairwise connections between FC_fMRI_ and FC_EEG_ exist, when relating each of the functional modalities to diffusion MRI (dMRI) connectivity (Wirsich et al., 2017). Specifically, when trying to explain the underlying structural connectivity by using the combination of fMRI- and EEG-derived FC, we observed that EEG-delta connectivity provides additional information to fMRI connectivity at the global level. Conversely, gamma contributes information beyond FC_fMRI_ locally in areas of the visual cortex. To date, the few existing investigations of concurrently recorded fMRI and source space EEG connectomes (Deligianni et al., 2014, 2016; Wirsich et al., 2017) remain correlational, and analysis of joint EEG-fMRI connectivity is largely unexplored. In consequence, the relationship between EEG and fMRI connectivity organization is still incompletely characterized.

Recently, Amico et al. (2017) have proposed applying independent component analysis (ICA) to whole-brain FC to extract independent components, also termed as independent connectivity traits. This connectivity ICA (connICA) framework has been shown to be useful in extracting the hybrid independent components jointly expressed across fMRI- and diffusion MRI (dMRI)–derived connectivity (Amico & Goñi, 2018a). An interesting feature of this approach is that it is able to identify hybrid joint connectivity components that are linked in terms of explaining subject-specific variance of spatially independent nonlinear relationships between both modalities. Note that in contrast to other bimodal ICA approaches (e.g., Eichele et al., 2009), hybrid connICA operates on connectivity values rather than on signal magnitudes or their time series. As such, the connICA approach provides the optimal framework to access multi-timescale connectome components. This is the case not only if the modalities contribute in a spatially consistent manner but also in a spatially divergent manner to the variance observed in different subjects.

In this study, we first asked if there exist spatially independent brain network patterns co-occurring over modalities in simultaneous EEG-fMRI data. Second, we were interested in determining how many independent connectivity patterns are collapsed into the global connectivity organization provided by FCs_fMRI_ and FCs_EEG_. And third, we sought to determine the contribution of different oscillation frequencies to the EEG connectivity organization, and how this frequency distribution relates to fMRI connectivity.

In summary, in this study we sought to answer if there exist spatially independent frequency-specific FC patterns co-expressed between modalities across subjects by extending the hybrid connICA approach proposed in (Amico & Goñi, 2018a) to concurrent EEG-fMRI resting-state data.

## METHODS

We use two independent datasets acquired from two different sites. The main dataset consisted of 26 healthy subjects with 3 runs of 10 minutes of concurrent EEG-fMRI during task-free resting state (Sadaghiani et al., 2010). The generalization dataset consisted of 14 subjects, 20 minutes resting-state runs of concurrent EEG-fMRI (Wirsich et al., 2017). The data was used to extract the main joint independent components co-occurring during EEG-fMRI resting state.

### Data Acquisition and Processing

#### I.a. Main dataset

##### Subjects and data of main dataset.

Magnetic resonance (MR) was acquired in 26 healthy subjects (8 females, mean age 24.39, age range 18–31) with no history of neurological or psychiatric illness. Ethical approval has been obtained from the local Research Ethics Committee (Comité de Protection des Personnes (CPP) Ile de France III), and informed consent has been obtained from all subjects.

Three runs of 10-min eyes-closed resting state were acquired in one concurrent EEG-fMRI session (Tim-Trio 3T, Siemens, 40 slices, TR = 2.0 s, 3.0 × 3.0 × 3.0 mm, TE = 50 ms, field of view 192, FA = 78°). EEG was simultaneously recorded using an MR-compatible amplifier (BrainAmp MR, sampling rate 5 kHz), 62 electrodes (Easycap), referenced to FCz, 1 ECG electrode, and 1 EOG electrode, while the scanner clock was time locked with the amplifier clock (Mandelkow et al., 2006). An anatomical T1-weighted MPRAGE (176 slices, 1.0 × 1.0 × 1.0 mm, field of view 256, TR = 7 min) was equally acquired. The acquisition was part of a study with two naturalistic film stimuli of 10 min not analyzed in this study (acquired after runs 1 and 2 of the resting state as described in Morillon et al., 2010). Subjects wore earplugs to attenuate scanner noise and were asked to stay awake, avoid movement, and close their eyes during resting-state recordings. Because of insufficient EEG quality in three subjects, one of three rest sessions was excluded.

##### Brain parcellation of main dataset.

T1-weighted images were processed with the Freesurfer suite (recon-all, v6.0, http://surfer.nmr.mgh.harvard.edu/) performing nonuniformity and intensity correction, skull stripping, and gray/white matter segmentation. The cortex was parcellated into 148 regions according to the Destrieux atlas.

##### fMRI processing of main dataset.

The fMRI time series were subjected to time slicing followed by spatial realignment using the SPM12 toolbox (revision 6906, http://www.fil.ion.ucl.ac.uk/spm/software/spm12). The subjects’ T1 image and Destrieux atlas were coregistered to the fMRI images. Average cerebrospinal fluid (CSF) and white matter signal from manually defined regions of interest (5-mm sphere, Marsbar Toolbox 0.44, http://marsbar.sourceforge.net) were extracted and were regressed out of the BOLD time series along with six rotation and translation motion parameters and global gray matter signal. Time series were bandpass filtered at 0.009–0.08 Hz (Power et al., 2014) and scrubbed using frame wise displacement (threshold 0.5) defined by Power et al. (2012). The Pearson correlation between all region pairs’ remaining time courses were used to build a functional connectivity matrix (FC_fMRI_).

##### EEG processing of main dataset.

The gradient artifact induced by the scanner on the EEG signal was removed using the template subtraction and adaptive noise cancellation followed by low-pass filtering at 75 Hz, downsampling to 250 Hz (Allen et al., 1998). Then, a cardiobalistic artifact template subtraction (Allen et al., 2000) was carried out using EEGlab v.7 (http://http://sccn.ucsd.edu/eeglab) and the FMRIB plug-in (https://fsl.fmrib.ox.ac.uk/eeglab/fmribplugin/). Data was then analyzed with Brainstorm software (Tadel et al., 2011), which is documented and freely available under the GNU general public license (http://neuroimage.usc.edu/brainstorm, version August 2017). Data was bandpass filtered at 0.3–70 Hz and segmented according to the TR of the fMRI acquisition (2-s epochs, based on a window length established by Colclough et al., 2016, and Wirsich et al., 2017). Epochs that contained head motion artifacts in EEG were visually identified and removed after semiautomatically preselecting epochs where signal in any channel exceeded the mean channel time course by 4 std.

Electrode positions were manually coregistered to the T1 image, and a forward model of the skull was calculated using the T1 image of each subject using the OpenMEEG BEM model (Gramfort et al., 2010; Kybic et al., 2005).

**Figure 1.**
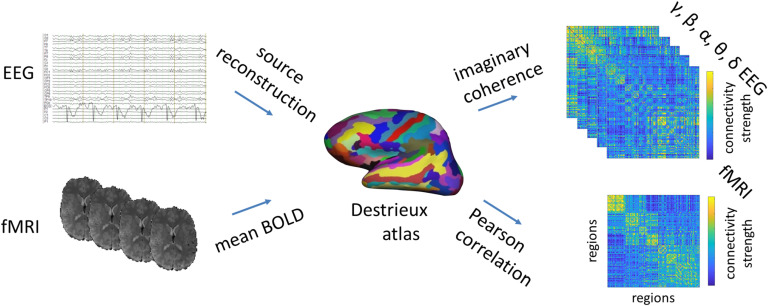
Construction of EEG and fMRI connectomes. EEG and fMRI data were parcellated into the 148 cortical regions of the Destrieux atlas as follows. EEG was source reconstructed to a fine-grained grid, and the time courses of the solution points were averaged per region and per subject. For fMRI, the BOLD signal time course was averaged over the voxels in each region for each subject. Pearson correlation of the mean fMRI-BOLD time courses and imaginary part of the coherency of averaged EEG source signals were used to build functional connectivity matrices/connectomes (FC_EEG_ and FC_fMRI_) for each subject.

EEG was re-referenced to the global average, and data were reconstructed into source space by using the Tikhonov-regularized minimum-norm with the Tikhonov parameter set to *λ* = 10% of maximum singular value of the lead field (Baillet et al., 2001). Source time series were averaged to the regions of the Destrieux atlas, and connectivity matrices were calculated for each segment by taking the imaginary coherency of delta, theta, alpha, beta, and gamma frequency bands between each region. Excluding the real part of coherency is a common method to avoid spurious connectivity stemming from volume conductance (Nolte et al., 2004). The final EEG functional connectivity (FC_EEG_) matrices were obtained by averaging the band-specific connectivity of all time segments (FC_δ_: 0.5–4 Hz; FC_θ_: 4–8 Hz; FC_α_: 8–12 Hz; FC_β_: 12–30 Hz; and FC_γ_: 30–60 Hz). All steps of EEG and fMRI connectome construction are summarized in Figure 1.

#### I.b. Generalization dataset

##### Subjects and data of generalization dataset.

MR was acquired in 14 healthy subjects (five females, mean age: 30.9, std: 8,6, Min/Max age: 20–55) with no history of neurological or psychiatric illness (see Wirsich et al., 2017). Ethical approval has been obtained from the local Research Ethics Committee (CPP Marseille 2), and informed consent has been obtained from all subjects.

A session of 21-min eyes-closed resting state was acquired using concurrent EEG-fMRI (Siemens Magnetom Verio 3T MRI-Scanner, 50 slices, TR = 3.6, 2.0 × 2.0 × 2.5 mm, TE = 27 ms, FA = 90°, a total of 350 volumes). EEG-fMRI were acquired during eyes-closed resting state, and the subjects were wearing a 64-channel EEG-cap (BrainCap-MR 3-0, Easycap, Hersching, Germany, according to the 10–20 system with one ECG channel and a reference at the mid-frontal FCz position). EEG was simultaneously recorded using an MR-compatible amplifier (BrainAmp MR, sampling rate 5 kHz), 63 electrodes (Easycap), referenced to FCz, and 1 ECG electrode, while the scanner clock was time locked with the amplifier clock (Mandelkow et al., 2006). An anatomical T1-weighted MPRAGE (TR = 1,900 ms, TE = 2.19 ms, 1.0 × 1.0 × 1.0 mm, 208 slices) was equally acquired. Subjects wore noise protection to attenuate scanner noise and were asked to stay awake, avoid movement, and close their eyes during resting-state recordings.

##### Brain parcellation of generalization dataset.

The parcellation procedure was identical to that of the main dataset (using Freesurfer 5.3).

##### fMRI processing of generalization dataset.

fMRI data processing was carried out as described in Wirsich et al. (2017). In brief, the data was processed as described above (using SPM12 6685, Marsbar 0.43). After regressing out movement, CSF, white matter and global gray matter signal, instead of bandpass filtering as used in the main dataset, fMRI data was filtered by wavelet analysis using the Brainwaver toolbox (version 1.6, http://cran.r-project.org/web/packages/brainwaver/index.html) (Achard et al., 2006; Wirsich et al., 2017). Equivalent to the main dataset, fMRI data points were scrubbed using framewise displacement (threshold 0.5) as defined by Power et al. (2012). The Pearson correlation of the remaining wavelet coefficients of scale two (equivalent to a frequency band 0.04–0.09 Hz) was used to build a functional connectivity matrix (FC_fMRI_).

##### EEG processing of generalization dataset.

EEG artifact correction was carried out using the Brain Vision Analyzer 2 software (Brain Products, Munich, Germany). To correct for the gradient artifact, a gradient template was subtracted followed by adaptive noise cancellation with 70-Hz low-pass filtering and downsampling to 250 Hz (Allen et al., 2000). Peaks from the ECG signal were extracted to generate a cardiac pulse artifact template averaged over the 100 last pulses. This template was then subtracted from the EEG signal (Allen et al., 1998). Eye movement was manually rejected using ICA and high-pass filtered at 0.3 Hz. Data was segmented into 3.6-s segments (according to one TR of the fMRI sequence and equivalent to Wirsich et al., 2017). Segments with obvious movement artifacts were manually excluded from the analysis. As in the main dataset, the remaining segments were then analyzed with Brainstorm software (Tadel et al., 2011), followed by FC_EEG_ matrix construction, with the difference that we used an older version of Brainstorm (January 2016 according to Wirsich et al., 2017).

#### Analytical methods

##### Hybrid connectivity ICA.

**Figure 2.**
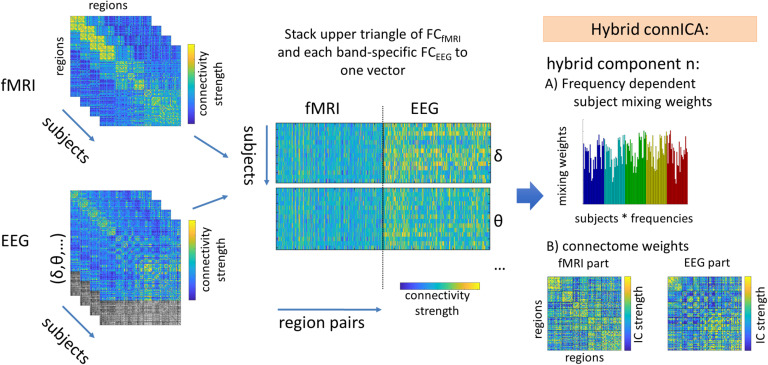
The upper triangular of each individual fMRI functional connectivity (FC_fMRI_) matrix (left, top) and lower triangular of each correspondent EEG functional connectivity (FC_EEG_) matrix (left, bottom) are added to a matrix where rows are the subjects times the EEG frequency bands and columns are their vectorized hybrid (fMRI-EEG) connectivity patterns; the fMRI vector is repeated and concatenated with each of the five canonical frequency bands (middle). The ICA algorithm extracts the n independent components (i.e., ICs) associated to the whole population and their relative ICA mixing weights across the subjects times the frequency bands (right).

We used the hybrid connICA (Amico et al., 2017; Amico & Goñi, 2018a), code available here: https://engineering.purdue.edu/ConnplexityLab/publications/connICA_hybrid_toolbox_v1.0.tar.gz) method as a data-driven way to disentangle the main brain network patterns underlying the FCs_fMRI_ and FCs_EEG_ (Figure 2).

To analyze the data in one hybrid vector, we uniformed the value distribution range of EEG (imaginary coherency original range 0 to 1) and fMRI (Pearson correlation, range −1 to 1) by transforming the EEG imaginary coherency according to the procedure in (Amico & Goñi, 2018a). In brief, we derived a correlation value from EEG by taking the Pearson correlation between the i_th_ and j_th_ row of the imaginary coherency-based connectome. This procedure results in a new correlation matrix where the values −1 and 1 indicate if the EEG nodes are connected antagonistically to the network or have high similarity (see also matching index in Rubinov & Sporns, 2010).

Then, for each subject, we first computed the concurrent FCs_fMRI_ and FCs_EEG_ at different frequency bands (δ, θ, α, β, and γ). We then stacked the upper triangular parts of each individual FC_fMRI_ together with the FC_EEG_ of each frequency band (FC_fMRI_ was repeated 5 times for each combination with one EEG frequency band) and added it to a matrix where rows are number of subjects times the number of frequencies, and columns are their bimodal connectivity pattern (see scheme in Figure 2). Second, we applied a principal component analysis (PCA) dimensionality reduction followed by an ICA run. As both PCA reduction and number of selected independent components (ICs) are an arbitrary choice, parameters were explored for keeping the principal components (PCs) that explain 75% to 90% of the data (in 4 steps of 5% each) and reducing the data from 5 to 20 ICs (in 4 steps of 5 ICs). The ICA algorithm was run (500 times, similarly to Amico & Goñi, 2018a) to extract the main hybrid (joint FC_EEG_-FC_fMRI_) components associated with the whole population. Third, the most robust components were selected. Here, robust is defined as appearing at least in 75% of ICA iterations with correlation higher than 0.75 for both IC connectivity strength and subject mixing weights. This approach has been shown to generate robust connICA estimation (Amico et al., 2017).

**Figure 3.**
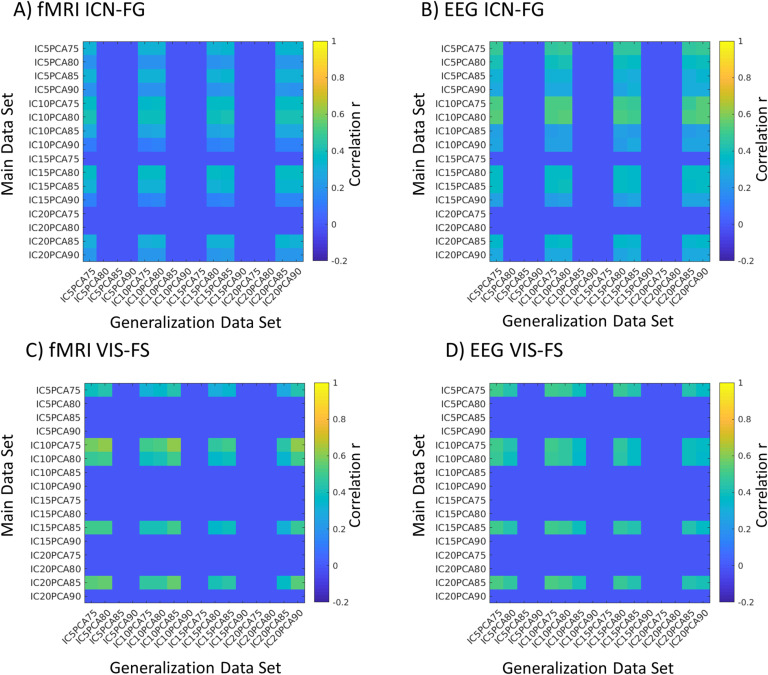
Impact of the choice of free parameters on the consistency across datasets. Matrices visualize the intercorrelation between the IC strengths of the two datasets by using different parameter sets, shown separately for the EEG and fMRI parts of the ICN-FG and VIS-FS components (A–D). We tested all combinations, keeping principal components with 75–90% of the variance, followed by an independent component analysis keeping 5–20 ICs (e.g., IC5PCA75 depicts an analysis using 5 ICs and keeping all PCs that cumulatively explain 75% of the data variance). Rows with consistent correlation of zero mean that in one of the datasets, the preceding separate ICC procedure had found no component passing stability requirements for this parameter combination. There is significant (*p* <10^−100^) spatial correlation of IC matrices between datasets for all parameters for which stable ICs had been identified (average correlation for the ICN-FG component: fMRI part *r* = 0.27; EEG part *r* = 0.35; and for the VIS-FS component: fMRI part *r* = 0.45; EEG part *r* = 0.44).

Two consistent hybrid ICs were observed across the majority of parameter pairs (the above described PCA reduction followed by limiting the number of ICs) and across both datasets. We chose the parameters by maximizing the correlation of the upper triangle of those two ICs between the two datasets (this is the maximum value when averaging the four diagonals in Figure 3; for across-parameter stability of the components *within* each dataset see Supporting Information Figures S1 and S2). Choosing separate parameters for each dataset can result in slightly higher correlation of ICs across datasets (i.e., keeping PCs that explain 75% of the variance in one dataset and 80% of the variance in the other; see Figure 3 off-diagonals and Supporting Information Table S1). However, in order to avoid overestimation of the correlation between the two datasets, we chose the same parameter configuration for both main and generalization datasets. For some parameter pairs we also observed that in the main dataset, stable components sometimes split up into two components. Both components are correlated to one component in the generalization dataset and can optionally be merged into one component that correlates even better with the component found in the generalization data. In case we found two components in the main dataset, we kept only the component that correlated more with the generalization dataset (to conservatively avoid adding an additional merging step). Finally, we kept the IC configuration most consistent between the two datasets: the first PCA components explaining 75% of the variance (12 first PCs in case of the main dataset and 14 first PCs for the generalization dataset) were kept for further analysis.

##### Intraclass correlation of mixing weights.

To characterize the properties of the resulting hybrid ICs, we assessed graph modularity and intraclass correlation (ICC; Bartko, 1966) of both EEG frequency-specific and subject-specific mixing weights. ICC is a measure to define the percent of agreement between units (ratings) of different groups (raters) (McGraw & Wong, 1996). The stronger the agreement between raters, the higher the ICC value. Here, we used ICC to define either the EEG frequency band weighting or the subject weighting of the IC mixing matrix as raters. With this approach we assess if the IC mixing matrix is separable by either EEG frequency or subject weight.

##### Modularity of ICs.

Modularity of EEG and fMRI IC matrices were calculated based on the Brain Connectivity Toolbox (version 2019_03_03, https://sites.google.com/site/bctnet/; Rubinov & Sporns, 2010). To test in how far the networks overlap with canonical ICNs (as proposed by Amico et al., 2017), we calculated modularity based on a predefined module label M for each region derived from the Yeo7 ICN networks (Yeo et al., 2011). Using this predefined set of modules enables us to interpret the observed modularity values as a measure of convergence with the well-known canonical ICN organization. For the calculation of modularity Q, negative and positive connectivity values were treated separately. Connectivity values were treated according to the negative asymmetry implementation proposed by Rubinov and Sporns (2011), weighting the modularity (Newman, 2004) of positive connections more than negative connections (see implementation in function community_louvain.m of the Brain Connectivity Toolbox):

$$Q=\frac{1}{v^{+}}\underset{i,j}{\sum }\left(w_{i}{,}_{j}^{+}-e_{i}{,}_{j}^{+}\right)\delta_{M_{i}M_{j}}-\frac{1}{v^{+}+v^{-}}\underset{i,j}{\sum }\left(w_{i}{,}_{j}^{-}-e_{i}{,}_{j}^{-}\right)d_{M_{i}M_{j}}$$ (1)

where the edge weight $w_{i}{,}_{j}^{+}$ = *w*_*i*,*j*_ in case the value is positive and $w_{i}{,}_{j}^{+}$ = 0 in case the value is negative, while $w_{i}{,}_{j}^{-}$ = *w*_*i*,*j*_ in case the value is negative and $w_{i}{,}_{j}^{-}$ = 0 in case the value is positive. *δ*_*M*_*i*_*M*_*j*__ = 1 is assigned in case both nodes are in the same module, and *δ*_*M*_*i*_*M*_*j*__ = 0 in case the nodes are in different modules M. $e_{i}{,}_{j}^{\pm }=\frac{\underset{i}{\sum }\left(w_{i}{,}_{j}^{\pm }\right)\underset{j}{\sum }\left(w_{i}{,}_{j}^{\pm }\right)}{v^{\pm }}$ with *v*^±^ = $\underset{i,j}{\sum }\left(w_{i}{,}_{j}^{\pm }\right)$ denotes the expected density of positive or negative weights, respectively (Rubinov & Sporns, 2011).

### Data availability

ConnICA code is available at https://engineering.purdue.edu/ConnplexityLab/publications/connICA_hybrid_toolbox_v1.0.tar.gz. Processed EEG and fMRI connectomes are available at OSF (https://osf.io/hberu/). Raw EEG-fMRI data is available on request.

## RESULTS

We observed correlations of connection strength for fMRI versus δ/θ/α/β/γ of *r* = 0.34/0.34/ 0.33/0.36/0.29 in the main dataset (replication dataset correlation were fMRI vs. δ/θ/α/β/γ: *r* = 0.34/0.32/0.33/0.37/0.16 as reported in Wirsich et al., 2017). Next, we sought to move beyond the prior correlational approaches by using joint fMRI-EEG decomposition of brain FC.

### Outcome of connICA After Determination of Free Parameters

When applying the connICA method to the EEG-fMRI connectivity matrices, as a function of the choice of the free parameters in the framework (PCA reduction and number of selected ICs; cf. Figure 3) we observed two to four stable reoccurring components in the main dataset, whereas two stable components reoccurred in the generalization dataset (using PCs explaining 75% of the variance followed by an ICA with 10 components; see Methods section).

**Figure 4.**
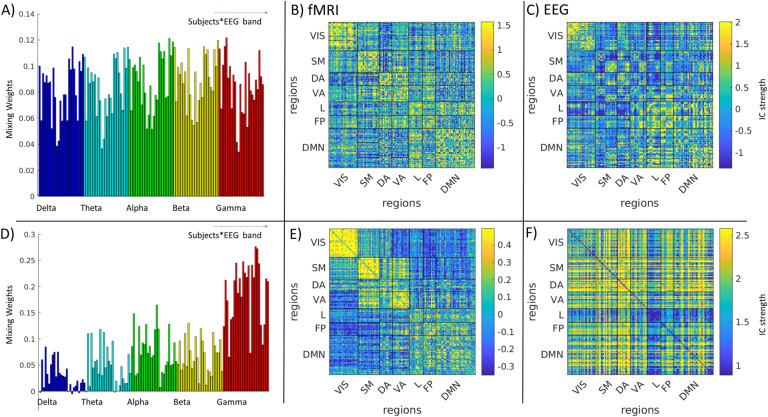
(A) Subject- and band-specific mixing weights of the hybrid EEG-fMRI ICN-FG component. (B) fMRI part of the ICN-FG component. (C) EEG part of the ICN-FG component. Note that as we stacked up all frequency-specific EEG connectomes for each subject (cf. Figure 2), we obtained a single EEG component part (C) associated with an ICA mixing weight for each subject and frequency (represented by one bar each in A). (D–F) These panels visualize equivalent aspects for the VIS-FS component. All panels represent the main dataset (for generalization data see Supporting Information Figure S3. Colorbars have been saturated at 95th and 5th percentile for better comparison with Figures 5 and 6). VIS = visual; SM = somatomotor; DA = dorsal attention; VA = ventral attention; L = limbic; FP = fronto-parietal; DMN = default mode network.

**Figure 5.**
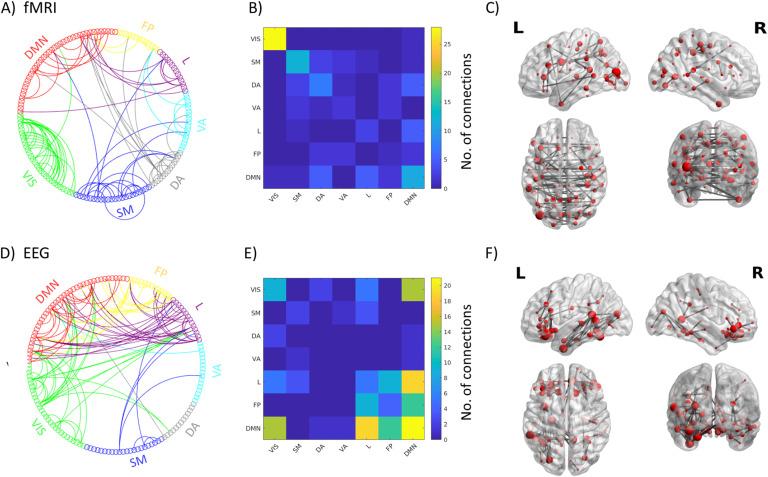
Connections with highest nodal strength (99th percentile) for the ICN-FG component: circle graphs show all connections between different ICN networks for fMRI (A) and EEG (D); matrices summarize the number of connections falling into each ICN-ICN pair for fMRI (B) and EEG (E); brain renderings show strongest connections of the components on a canonical reconstructed cortical surface for the fMRI (C) and EEG (F) part of the hybrid component. Data correspond to the main dataset; for the results of the generalization dataset see Supporting Information Figure S4. VIS = visual; SM = somatomotor; DA = dorsal attention; VA = ventral attention; L = limbic; FP = fronto-parietal; DMN = default mode network.

**Figure 6.**
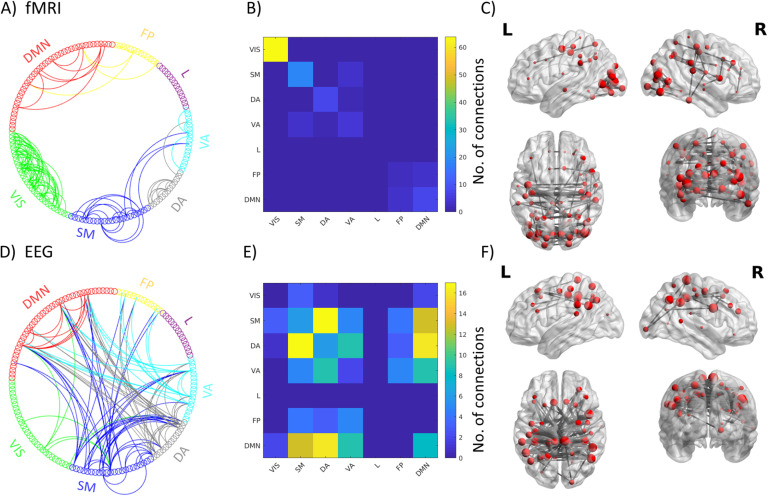
Connections with highest nodal strength (99th percentile) for the VIS-FS component: circle graphs show all connections between different ICN networks for fMRI (A) and EEG (D); matrices summarize the number of connections falling into each ICN-ICN pair for fMRI (B) and EEG (E); brain renderings show strongest connections of the components on a canonical reconstructed cortical surface for the fMRI (C) and EEG (F) part of the hybrid component. Data correspond to the main dataset; for the results of the generalization dataset see Supporting Information Figure S5. VIS = visual; SM = somatomotor; DA = dorsal attention; VA = ventral attention; L = limbic; FP = fronto-parietal; DMN = default mode network.

Two stable components were appearing in both datasets (Figure 3, Supporting Information Figures S1 and S2):

1.  We observed an Intrinsic Connectivity Network-Frequency-General component (ICN-FG) that captures all the main within-network connectivity in the ICNs described by Yeo et al. (2011) (Figure 4 and Figure 5). The mixing weights do not differ for different frequencies (Figure 4A).
2.  We observed a Visual-Frequency-Sensitive component (VIS-FS) that shows two different patterns for fMRI and EEG that jointly co-occur. The fMRI part mainly captures the connectivity within the visual network (VIS) and the connectivity between visual and somatomotor (SM) networks (Figure 4 and Figure 6), whereas the EEG part captures mainly connectivity between ICNs. Mixing weights differ according to frequency band Figure 4D).

### The ICN-Frequency-General Component

The ICN-FG component was identified in both the main and generalization datasets, with spatial similarity across datasets observed for both the fMRI IC weight matrix (i.e., the spatial pattern in Figure 4B compared to that in Supporting Information Figure S3; *r* = 0.36, *p* < 1.0^−300^) and the EEG IC weight matrix (Figure 4C vs. Supporting Information Figure S3C; *r* = 0.51, *p* < 1.0 ×10^−300^). Lifting our requirement of keeping parameters identical across the two datasets would further improve the correlations across datasets (cf. off-diagonals in Figure 3); for the fMRI part, the correlation of IC weights would be maximized to *r* = 0.41 (*p* = <1.0 ×10^−300^) in case of a PCA selecting 75% of the variance followed by an ICA limited to 10 ICs (IC10PCA75) for the main dataset, while using IC10PCA80 for the generalization dataset. Conversely, the correlation would be minimized to *r* = 0.12, (*p* = 4.4 ×10^−34^) by combining IC10PCA90 (main) vs. IC5PCA75 (generalization). The mean correlation between all possible combinations of parameters was 0.27 (*p* = 5.5 ×10^−183^). For the EEG part, the IC weight correlation is maximized to *r* = 0.54 (*p* = <1.0 ×10^−300^) when combining IC10PCA80 (main) with IC20PCA90 (generalization), and minimized to *r* = 0.22 (*p* = 3.8 ×10^−123^) for IC10PCA85 (main) combined with IC20PCA85 (generalization). The mean cross-dataset correlation over all parameter combinations was *r* = 0.35 (*p* = <1.0 ×10^−300^). This similarity confirms the robustness of the hybrid component across datasets and across a variety of parameter combinations.

On visual inspection, EEG and fMRI parts of the IC (Figure 4B and 4C) share partial spatial similarity. This is in line with small but significant correlation between EEG and fMRI parts of the ICN-FG independent component (main dataset: *r* = 0.11, *p* = 3.66 ×10^−30^; generalization dataset *r* = 0.37, *p* = 1.42 ×10^−251^). Assessed visually, both EEG and fMRI parts of the IC express a strong organization into ICNs. We empirically tested this observation by calculating the modularity of the network given the predetermined labels of the seven ICNs as defined by Yeo et al. (2011). The mapping of the ICN-FG component to the Yeo ICNs is visualized in Figure 5A, 5B, 5D, and 5E. For FC_fMRI_, we found significantly higher modularity than in randomized networks (5,000 iterations with preserved weighted degree distribution and weighted degree sequence) (Maslov & Sneppen, 2002; Rubinov & Sporns, 2011) (q_fMRI_ = 0.22, *p* < 0.0002; generalization q_fMRI_ = 0.37, *p* < 0.0002). Similarly, we found significant moderate modularity for FC_EEG_ (q_EEG_ = 0.093, *p* < 0.0002/generalization *q* = 0.14, *p* < 0.0002, same null model as ndom network with FC_fMRI_, 5,000 iterations). The significant modularity implies that connectivity is generally stronger within ICN modules than across them, confirming the relative spatial closeness of the hybrid component to the well-known ICN organization.

In addition to supporting the ICN backbone of the intrinsic connectivity architecture, the IC also showed a high subject-specific fingerprint on all its mixing weights (Figure 4A, ICC_subject_ = 0.85, *p* < 1.0 ×10^−10^; generalization ICC_subject_ = 0.48, *p* = 2.18 ×10^−6^), but importantly nonsignificant (main dataset, ICC_subject_ = −0.005, *p* = 0.42) or low (generalization dataset ICC = 0.27, *p* = 0.00023673) ICC for frequency category. This result shows that the ICN-FG component has a strong contribution from all timescales and is not frequency specific.

### The Visual-Frequency-Sensitive Component

The VIS-FS component was found in both datasets with spatial similarity across datasets of r = 0.50 (*p* <10^−300^; Figure 4E vs. Supporting Information Figure S3) for the fMRI IC matrix and *r* = 0.50 (*p* <10^−300^; Figure 4F vs. Supporting Information Figure 3F) for the EEG IC matrix. When permitting parameters to differ across the two datasets (off-diagonals in Figure 3), this cross-dataset correlation of the IC matrix is maximized for the fMRI part to *r* = 0.62 (*p* <10^−300^) by combining a PCA selecting 75% of the variance followed by and ICA limited to 10 ICs (IC10PCA75) for the main dataset and IC10PCA85 for the generalization dataset. The correlation is minimized to *r* = 0.27 (*p* = 5.5 ×10^−183^) for IC5PCA75 (main) and IC20PC85 (generalization). The mean correlation of all parameter configurations is *r* = 0.45 (*p* = <1.0 ×10^−300^). The correlation of the EEG component is maximized to *r* = 0.53 (*p* = <1.0 ×10^−300^) in case of combining IC20PCA85 (main) with IC5PCA75 (generalization) and minimized to *r* = 0.35 (*p* = <1.0 ×10^−300^) when combining IC10PCA80 (main) and IC20PCA90 (generalization). The mean cross-dataset correlation of all parameter configurations is *r* = 0.44 (*p* = <1.0 ×10^−300^). Again, this similarity confirms the robustness of the identified hybrid component across datasets.

Compared with the ICN-FG component, EEG and fMRI parts of the VIS-FS component diverge considerably in their spatial pattern (Figure 4E and 4F). In line with this divergence, EEG and fMRI matrices showed a very low but significant anticorrelation (main dataset *r* = −0.10, *p* = 1.09 ×10^−26^; generalization dataset r = −0.15, *p* = 5.79 ×10^−58^). The mapping of the VIS-FS component to the Yeo ICNs is visualized in Figure 6 (A, B, D, E). The fMRI pattern of this component is dominated by ICN organization (Figure 4E) similar to the observation in the ICN-FG component. Supporting this observation, fMRI again showed significant modularity when mapped to Yeo’s seven ICNs (compared to 5,000 iterations of random networks with preserved weight and strength distribution of FC_fMRI_/FC_EEG_; Rubinov & Sporns, 2011: q_fMRI_ = 0.32, *p* < 0.0002; generalization dataset q_fMRI_ = 0.30, *p* < 0.0002). However, the visual (VIS) ICN, and to a lesser degree the somatomotor (SM) ICN, are more strongly expressed than other ICNs. The EEG part (Figure 4F) co-occurring with the aforementioned fMRI pattern depicts a more diverse connectivity profile diverging from the ICN architecture. This divergence is confirmed by lack of significant modularity when mapped to the seven canonical ICNs (q_*EEG*_ = −0.012, *p* = 0.95; generalization q_*EEG*_ = −0.018, *p* = 0.97). The most salient characteristics of the EEG pattern are strong connectivity among default mode network (DMN), S, M and dorsal attention (DA) networks (Figure 4F and 6D and 6F).

Interestingly, these joint EEG-fMRI patterns are sensitive to different frequency bands under consideration. In other words, the mixing weights associated with the VIS-FS component change in magnitude as a function of frequency (Figure 4D, ICC_freq_ = 0.69, *p* < 1.0 ×10^−10^; generalization dataset ICC_freq_ = 0.48, *p* = 3.51 ×10^−8^), and do not represent a subject-specific fingerprint (ICC_subject_ = −0.11, *p* = 0.98, generalization dataset ICC_subject_ = 0.07, *p* = 0.19). The frequency sensitivity was characterized by considerably stronger mixing weights for the gamma band compared with other frequencies.

### Relationship Between Components and Head Movement

To test if subject-specific mixing weights are driven by head movement, we checked for a monotonous relationship between the mixing weights and mean framewise displacement (FD), as well as between mixing weights and the number of scrubbed volumes by using Spearman ranked sum correlation. For the ICN-FG component, we found an negative correlation between subject-specific mixing weights and motion across all bands in the main dataset but not in the generalization dataset (main dataset: FD vs. mixing weights (Figure 4A): rho = −0.44, *p* = 1.9 ×10^−7^; no. of scrubbed volumes vs. ICA: rho = −0.37, *p* = 1.4 ×10^−5^/generalization dataset: FD vs. mixing weights: rho = 0.17, *p* = 0.14; no. of scrubbed volumes vs. mixing weights: rho = 0.042, *p* = 0.73).

For the VIS-FS component, we did not find a relationship between subject-specific mixing weights and motion across any band in either dataset (main dataset: FD vs. mixing weights (Figure 4D): rho = 0.07, *p* = 0.40; no. of scrubbed volumes vs. ICA: rho = 0.12, *p* = 0.14/generalization dataset: FD vs. mixing weights: rho = 0.11, *p* = 0.36; no. of scrubbed volumes vs. mixing weights: rho = 0.04, *p* = 0.74). To summarize, we found a negative relationship between movement and mixing weights of the ICN-FG component in the main dataset. This outcome indicates that for the main dataset, subjects with movement contribute less to the ICN-FG component.

Of note, as mentioned in the Methods section, we observed a second component with similar characteristics to the ICN-FG component in the main dataset. This component did not show any relationship to head motion (see detailed analysis in Supporting Information Results section). This observation further supports that the characteristic features of the ICN-FG component are not dependent on motion.

## DISCUSSION

Since BOLD fMRI measures neural energy consumption, FC_fMRI_ collapses over a mixture of electrophysiological FC (FC_EEG_) mechanisms in various oscillatory frequency bands. To disentangle the complex relationship between FC_fMRI_ and multifrequency FC_EEG_, we applied a data-driven decomposition of bimodal connectomes for simultaneous EEG-fMRI recordings. Two robust spatially independent bimodal connectome components, each representing FC_fMRI_ and FC_EEG_, were found across all frequency bands and both independent datasets. The first component, which we call the Intrinsic Connectivity Network–Frequency-General component (ICN-FG), has a uniformly distributed frequency fingerprint and is linked to ICNs in both modalities (Figures 4A and 5). Conversely, the second component, which we call the Visual–Frequency-Sensitive component (VIS-FS), spatially diverges between EEG and fMRI and is sensitive to different frequency bands under consideration (i.e., the mixing weights associated to the VIS-FS component change in magnitude as a function of frequency; see Figures 4D and 6).

### ICN-FG Component

The ICN-FG component captures the main within-network connectivity in well-known neurocognitive networks for EEG and fMRI. The EEG and fMRI patterns are co-expressed over subjects and quantified by subject-specific mixing weights. This result corroborates previous findings extracting the same robust ICN-FG components from different fMRI datasets (Amico et al., 2017; Contreras et al., 2017).

The fact that we found this ICN-conform stable hybrid component across modalities might suggest that the within-network connectivity dynamics are “scale-independent,” that is, to some extent, insensitive to whether one considers the millisecond-scale of the FC_EEG_ connectome in a specific frequency, or the infraslow-scale of the FC_fMRI_ connectome. Previous work on microstates have indeed demonstrated temporal scale-free behavior in EEG ranging from fast millisecond to slow second time range (Van de Ville et al., 2010). Such timescale invariance is also in line with more recent findings linking fMRI to EEG (Deligianni et al., 2014; Wirsich et al., 2017) and to MEG connectivity (Brookes et al., 2011; Colclough et al., 2016).

We found strong ICC at the subject level, suggesting a strong fingerprint for each subject (Amico & Goñi, 2018b). This result should be interpreted very carefully, however, as we observed in the main dataset (but not in the replication dataset) that those subject-specific mixing weights are negatively correlated with head movement for the ICN-FG component. As such, the subject-specific mixing weights should be interpreted as subject-specific contributions to the hybrid component that might also be weighted by session-specific individual conditions such as vigilance state and movement of the subject. This possibility also implies that the final connectivity component is “corrected” for those subject-specific (linear) contributions, consequently representing a clean component common among all subjects. As we showed in the main dataset, the ICN-FG component can split into two parts, one being anticorrelated to movement and one being uncorrelated to movement. This demonstrates the ability of ICA to decompose the connectivity matrix into behaviorally relevant parts provided a sufficient number of subjects (the larger main dataset of 26 subjects was able to detect a split of the ICN-FG component that could not be observed in the smaller generalization dataset).

In conclusion, the ICN-FG component suggests that the presence of strong electrophysiological communication in all frequencies (widely distributed, with ventral dominance) contributes to strong ICN architecture in fMRI.

### VIS-FS Component

The VIS-FS hybrid component shows two spatially divergent patterns for fMRI and EEG (Figure 4C and 4F). The fMRI part mainly captures the connectivity within visual network (VIS) and the connectivity between visual and somatomotor (SM) networks but also other ICNs. On the other hand, the EEG part co-occurring with the aforementioned fMRI pattern depicts a more diverse connectivity profile that captures between-network connectivity, especially between DMN. Although the pattern has not been described before, it reproduces across both datasets (correlation between EEG IC strength of the two datasets *r* = 0.50; see Figure 3).

The observed difference across EEG and fMRI parts of the VIS-FS component suggests that the hybrid connICA is able to detect the complementary information carried over the two different timescales measured by FC_fMRI_ and FC_EEG_. The fMRI IC weights of the component are in line with previous findings extracting VIS-SM independent components from different fMRI datasets (Amico et al., 2017; Contreras et al., 2017). On the other hand, the γ-band-driven electrophysiological FC VIS-FS component is in line with the results of Wirsich et al. (2017) who observed a global contribution of delta but a contribution of gamma to predicting diffusion MRI connectivity specifically in visual areas. The results reported here are consistent with this pattern, as the VIS-FS component is mainly driven by gamma oscillations, whereas delta does not contribute to this component and only contributes to the global pattern of the ICN-FG component (see Figure 4A).

The dominance of the gamma band in this component tied with an occipitally focused fMRI counterpart may be surprising in light of the well-known predominance of alpha power in occipitoparietal cortex. Here, it is important to recall the difference between local power versus long-range coupling of electrophysiological oscillations. While the former is dominated by the alpha band, whole-brain oscillation-based connectivity in M/EEG paints a different picture (for an extensive review see Sadaghiani & Wirsich, 2019). In particular, early studies defining ICNs in MEG (Brookes et al., 2011; Hipp et al., 2012) found that alpha and beta bands provide the strongest connectivity in all observed ICNs (presumably due to higher SNR; cf. Hipp & Siegel, 2015) without a unique prevalence in VIS/DA networks. After accounting for SNR differences across frequencies, Hipp and Siegel (2015) computed a connection-wise measure of how strongly fMRI-based connectivity is tied to MEG-based amplitude coupling in each frequency (a measure of local homogeneity of the cross-modal relationship). They found that the relation between alpha coupling and hemodynamic correlations was particularly pronounced between frontal and temporal areas, concluding a deviation from the prominence of local alpha power in occipital and parietal areas. Similarly, no dominance of alpha has been reported for connections of the VIS network in concurrent EEG-fMRI studies (Deligianni et al., 2014; Wirsich et al., 2017). In sum, in contrast to local spectral power in visual cortex, neither phase coupling nor amplitude coupling of occipito-parietal connections appear to be stronger in the alpha compared with other frequency bands. In the following, we propose scenarios that may explain the pattern observed in the VIS-FS component.

### Mutual Versus Divergent Contributions of Hemodynamic and Electrophysiological Recordings

The here-observed duality of spatially mutual and discrepant contributions (ICN-FG and VIS-FS components, respectively) of hemodynamic and electrophysiological recordings has also been discussed in task-paradigm studies. Taking face recognition tasks as an example, mutual contributions of EEG and fMRI have been readily observed (Walz et al., 2013; Wirsich et al., 2014). However, face recognition tasks also engage discrepant contributions across the two modalities; it has been shown that while the face-selective N170 originating from the fusiform gyrus in MEG is not modulated by attention, fMRI show a strong modulation in the same region (Furey et al., 2006).

Such divergence across modalities speaks to the view that M/EEG and fMRI may in part capture preferentially different types of neural activity, rather than considering the fMRI signal merely a temporally blurred integration over electrophysiological activity. For example, unlike M/EEG, modulatory processes (such as states of motivation, attention and memory) may often dominate the hemodynamic signal (Logothetis, 2008). More recently, Hari and Parkkonen (2015) have suggested that M/EEG most strongly weight fast-conducting pathways (thick myelinated fibers), while the main contribution to the BOLD signal comes from neuronal ensembles connected via slow fiber pathways (densely packed small fibers). Applying this viewpoint to the VIS-FS component, the fMRI part would be more heavily driven by slow-conducting pathways, while the EEG part would more strongly reflect fast connections. In this scenario, the divergent topography of the EEG and fMRI parts fits well with the fact that slow-conducting fibers are more prevalent in local connections, while the fast-conducting fibers support long-distance connections (Aboitiz et al., 1992; Ringo et al., 1994). Specifically, the fMRI part of the VIS-FS component comprises more local within-ICN connections (especially for VIS and SM, which are least distributed), as apparent in significant graph modularity. Conversely, the EEG part of the VIS-FS component is foremost reflective of cross-ICN and, therefore, longer connections.

The co-occurrence of these two aspects within the VIS-FS hybrid component would then imply that fast-conducting pathways distributed broadly between ICNs and operating primarily in the gamma band (captured by EEG) would be linked to slow-conducting pathways primarily within ICNs, especially VIS and SM (captured by fMRI). These features should thus be considered as intertwined characteristics of the connectome. Another implication is that gamma, the fastest electrophysiological oscillation band, plays a special role over and above other bands in fast-conducted inter-ICN connectivity, which might be linked to cortical layer–specific preferences of the gamma band (Scheeringa & Fries, 2017; Scheeringa et al., 2016). In short, a partial preference of EEG and fMRI for nonoverlapping classes of pathways could explain how a spatially divergent connectivity pattern could be tied across the two modalities in the VIS-FS component. To summarize, the observation of two robust bimodal components, one with convergent and one with divergent topography across EEG and fMRI (ICN-FG and VIS-FS, respectively), would support the view that the two data modalities capture partly overlapping and partly independent neural activity.

From a systems neuroscience point of view, the concurrent convergence and divergence of multimodal connectivity patterns reflect recent findings of computational modeling of scale-free dynamics. In line with the convergent aspect, a large body of literature is showing that computational models of FC generated from structure show a common pattern across several timescales (Deco, Jirsa, McIntosh, Sporns, &Kötter, 2009, 2013; Ghosh et al., 2008; Hansen et al., 2015; Honey et al., 2007). This speaks to the ICN-FG component and its frequency independence observed in our study, likewise showing a common component across all observed timescales. Conversely, speaking to the divergent aspect, some evidence suggests additional timescale–specific contributions to FC organization (for discussion see Sadaghiani & Wirsich, 2019, section 3a). Specifically, recent trimodal and modeling studies demonstrate that EEG provides complimentary information to that obtained from fMRI when linking structural to FC (Schirner et al., 2018; Wirsich et al., 2017). These findings are in line with the above-discussed hypothesis that electrophysiological and hemodynamic signals may in part be associated with different fibers (Hari & Parkkonen, 2015). It has been recently demonstrated that tuning free parameters of a computational model for each region as compared to a global whole-brain level improves the link between structural and FC (Wang et al., 2019). This local modification of parameters permits local variation of FC timescales in line with the frequency sensitivity of the VIS-FS component observed in our study. This observation further highlights the complimentary information of FC on several timescales not predicted by the earlier computational models but likely harnessed by our bimodal connICA.

### Methodological Considerations, Limitations, and Future Directions

Like in any data-driven approach (and in rsfMRI in general) preprocessing steps remain arbitrary. In particular, here the parameters of PCA-based variance reduction and number of final ICs can slightly change the results. We tried to avoid this parameter-dependence by fitting the optimal parameter to the maximum correlation between two independent datasets. Nevertheless, the analysis design still remains flexible and parameter sets most likely also depend on SNR of any given dataset (recording length, field strength of the MR scanner). Also, the selection criteria for stable ICAs defined by (Amico et al., 2017) remain arbitrary, and further work should explore the parameters of the stability criteria or use other approaches to define ICA stability (e.g., an adequate null model).

Another important parameter might be the number of subjects used in the approach. The here-used generalization dataset had only 14 subjects with a resting-state acquisition of 20 min (as compared to the 26 subjects of the main dataset with each 30-min total of resting-state acquisition). Note that this relatively small number of subjects might result in an overfitting of the ICA weights to overly general features ignoring more detailed features. As shown in Figure 3 (and Supporting Information Figures S1 and S2), the ICN-FG component is detectable for more parameter configurations in the main dataset than in the generalization dataset (13 vs. 7 configurations). This suggests that it is easier to extract stable ICs when more data points/subjects are available. Note, however, that this was not true for the VIS-FS component (we found five configurations for the main dataset, whereas the generalization dataset had nine stable configurations). It remains unclear if increasing the sample size will help to solve this variability of the ICA approach (see Amico & Goñi, 2018a, and Supporting Information).

These constraints notwithstanding, the bimodal connICA approach provides novel information complementing those obtained from other methodologies. Deligianni et al. (2014) predicted EEG from fMRI and vice versa using canonical correlation analysis. Another approach would be to link multimodal connectomes by using partial least squares (Mišić et al., 2016). As compared to those correlational approaches, the choice of using ICA follows the general assumption that static connectivity holds *independent* mixed connectivity signals (Amico et al., 2017). The informativeness of such ICs has, for example, been established through their association with levels of consciousness (Amico et al., 2017), structural connectivity (Amico & Goñi, 2018a), and cognitive status in Alzheimer’s disease (Contreras et al., 2017). As such, the analyses applied here provide a data-driven approach to extract independent hybrid connectomes beyond correlational approaches.

### Conclusion

In conclusion, this work sheds new light on the relationship between EEG and fMRI connectivity, suggesting that parts of the joint EEG-fMRI resting-state connectivity are related across timescales and modalities in a spatially independent manner. This data-driven approach based on hybrid extraction of independent connectivity components shows great potential for future research in the field, especially for simultaneous EEG-fMRI in clinical populations.

## ACKNOWLEDGMENTS

We thank Katia Lehongre and Benjamin Morillon (main dataset) and Maxime Guye and Jean-Philippe Ranjeva (generalization dataset) for generously sharing their data.

## SUPPORTING INFORMATION

Supporting Information for this article is available at https://doi.org/10.1162/netn_a_00135.

## AUTHOR CONTRIBUTIONS

Jonathan Wirsich: Conceptualization; Data curation; Formal analysis; Investigation; Methodology; Software; Validation; Writing - Original Draft; Writing - Review & Editing. Enrico Amico: Conceptualization; Investigation; Methodology; Software; Writing - Original Draft; Writing - Review & Editing. Anne-Lise Giraud: Data curation; Funding acquisition; Resources; Writing - Review & Editing. Joaquín Goñi: Funding acquisition; Methodology; Supervision; Writing - Original Draft; Writing - Review & Editing. Sepideh Sadaghiani: Data curation; Funding acquisition; Investigation; Project administration; Supervision; Writing - Original Draft; Writing - Review & Editing.

## FUNDING INFORMATION

Sepideh Sadaghiani, National Institutes of Health (http://dx.doi.org/10.13039/100000002), Award ID: R01MH11622601A1. Sepideh Sadaghiani, National Institutes of Health (http://dx.doi.org/10.13039/100000002), Award ID: R21NS10460302. Sepideh Sadaghiani, Beckman Institute for Advanced Science and Technology, University of Illinois, Urbana-Champaign (http://dx.doi.org/10.13039/100005471), Award ID: MoCC seed grant. Joaquín Goñi, National Institutes of Health (http://dx.doi.org/10.13039/100000002), Award ID: R01EB022574. Joaquín Goñi, National Institutes of Health (http://dx.doi.org/10.13039/100000002), Award ID: R01MH108467. Joaquín Goñi, Indiana Alcohol Research Center, Award ID: P60AA07611. Anne-Lise Giraud, European Research Council (http://dx.doi.org/10.13039/501100000781), Award ID: 260347.

## TECHNICAL TERMS

Functional connectivity (FC): Denoting the temporal dependency of signal time courses at the systems level d (e.g., from fMRI, EEG, or MEG) measured from distributed brain regions.
Connectome: A whole-brain map of structural or functional neural connectivity. At the systems level, connections are typically established among brain regions, e.g., of a brain atlas.
Intrinsic connectivity networks (ICNs): Networks that spontaneously exhibit temporal dependency among neural activity time courses of their distributed regions. Regions of a given ICN also co-activate in response to the same cognitive demands.
Independent component analysis (ICA): A computational method for disentangling the independent additive subcomponents of a multivariate input signal, under the assumption that the subcomponents are non-Gaussian signals and that they are statistically independent from each other.
Principal component analysis (PCA): A statistical procedure that uses an orthogonal transformation to convert a set of observations of possibly correlated variables into a set of values of linearly uncorrelated variables called principal components.
Intraclass correlation (ICC): An inferential statistic for quantitative measurements that are organized into groups. It describe show strongly units in the same group resemble each other. A typical application consists of the assessment of consistency or reproducibility of quantitative measurements made by different observers measuring the same quantity.
