# Supplementary material for: Multi-timescale hybrid components of the functional brain connectome: A bimodal EEG-fMRI decomposition
Source: Netw Neurosci. 2020 Jul 1;4(3):658–77. doi: 10.1162/netn_a_00135 (PMC7462430; doi:10.1162/netn_a_00135)
Supplement: Supplementary file 1 [file netn-04-658-s001.pdf]

Wirsich, J., Amico, E., Giraud, A., Goñi, J., & Sadaghiani, S. (2020). Supporting information for "Multi-timescale hybrid components of the functional brain connectome: A bimodal EEG-fMRI decomposition." *Network Neuroscience*, *4*(3), 658–677. https://doi.org/10.1162/netn_a_00135

Supplementary Material

SI Figures

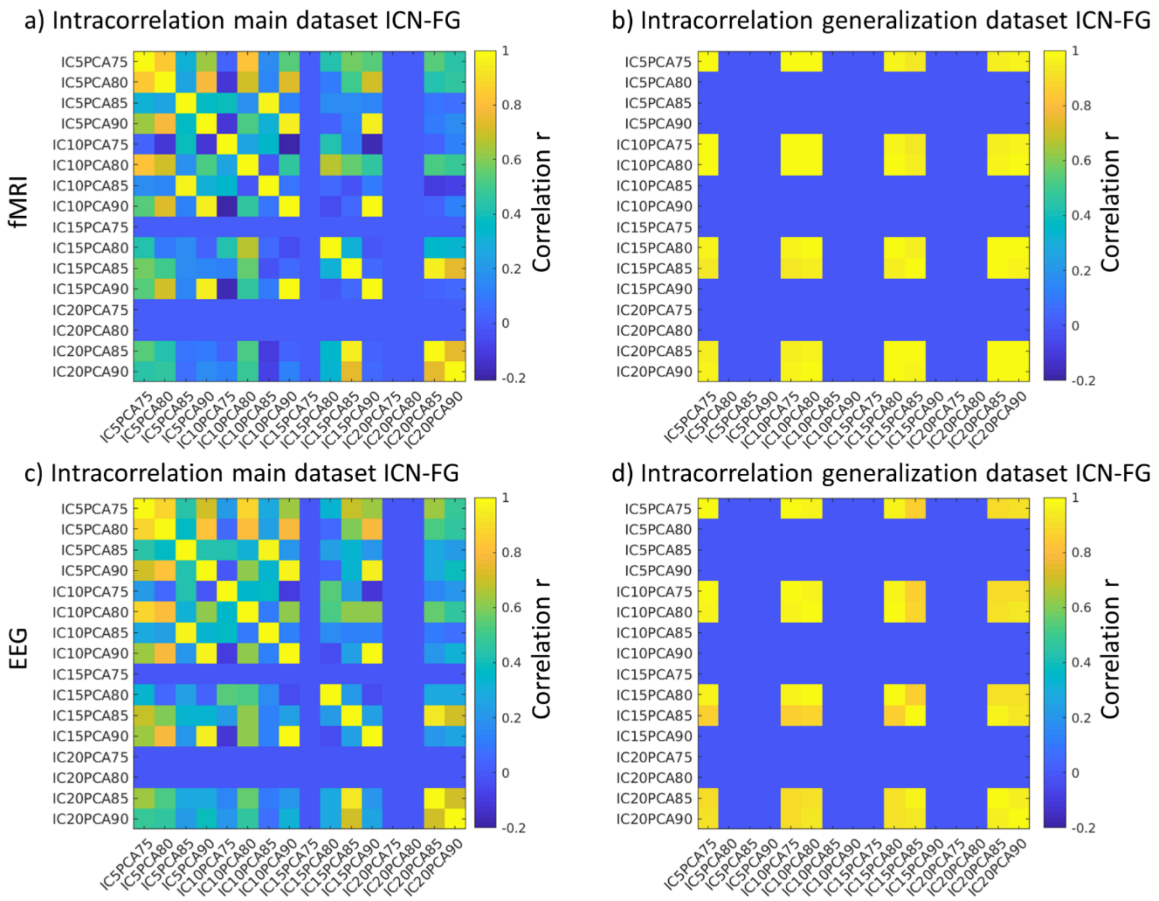

Figure SI1: Intracorrelation of the EEG and fMRI part of the IC strengths for ICN-FG component of the main dataset using different parameter sets. We tested all combinations keeping principal components with 75-80% of the variance followed by an independent component analysis keeping 5-20ICs (e.g. IC5PCA75 depicts an analysis using 5ICs and keeping all PCs explaining 75% of the data variance.). Rows with consistent correlation of zero mean that no ICN-FG component was found for this parameter combination.

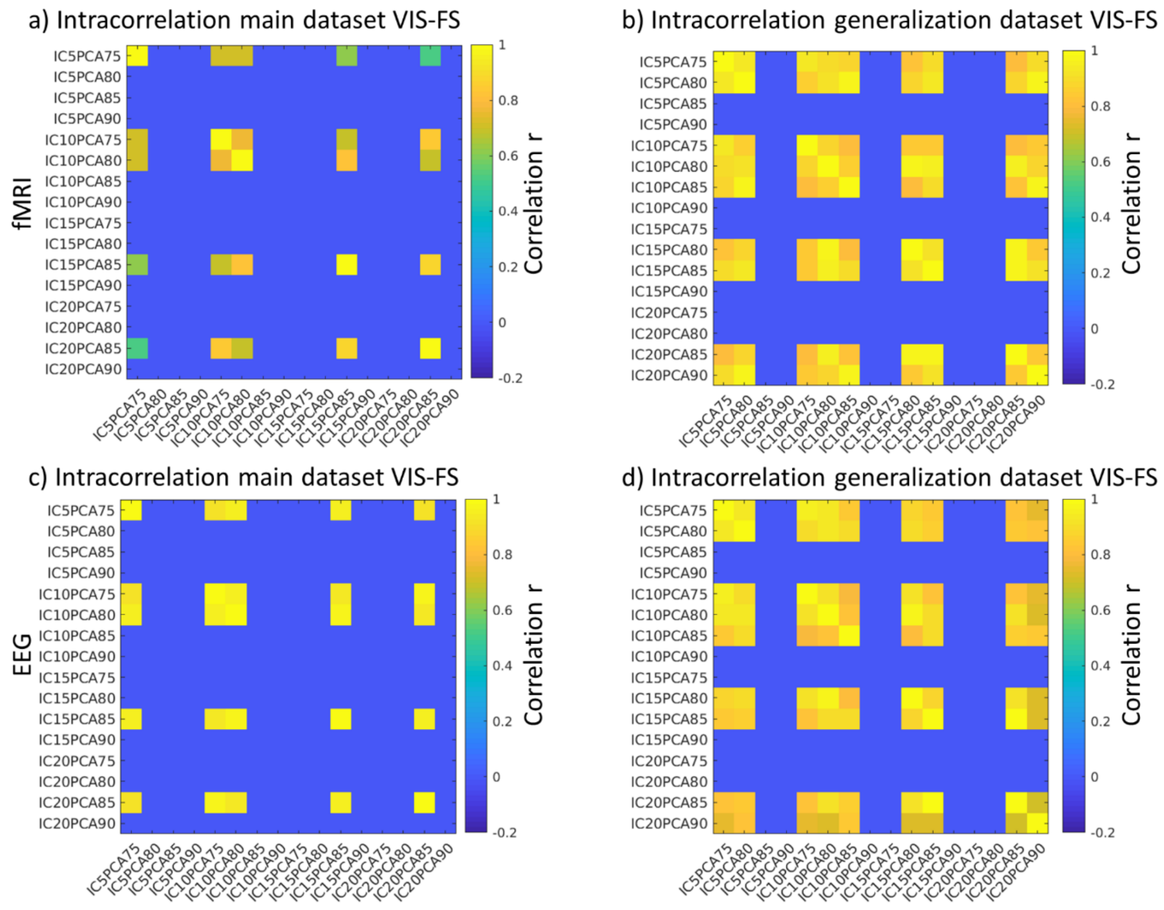

Figure SI2: Intracorrelation of the EEG and fMRI part of the IC strengths for VIS-FS component of the main dataset using different parameter sets. We tested all combinations keeping principal components with 75-80% of the variance followed by an independent component analysis keeping 5-20ICs (e.g. IC5PCA75 depicts an analysis using 5 ICs and keeping all PCs explaining 75% of the data variance.). Rows with consistent correlation of zero mean that no VIS-FG component was found for this parameter combination.

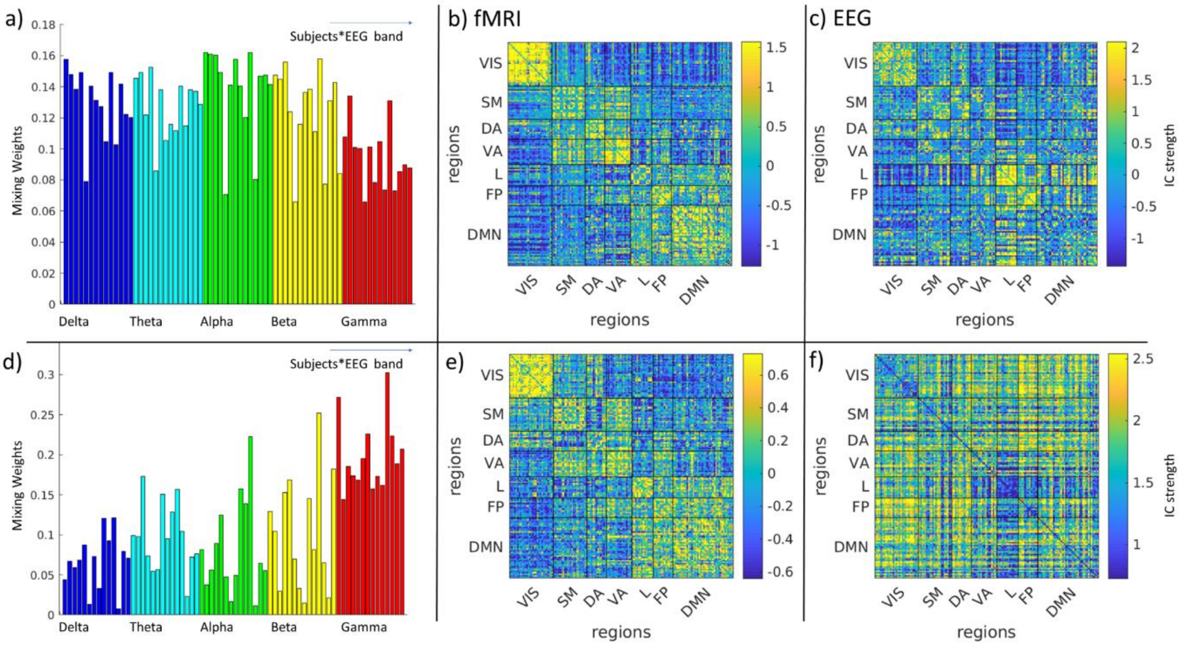

Figure SI3: a) Subject- and band-specific mixing weights of the hybrid EEG-fMRI ICN-FG component. b) fMRI part of the ICN-FG component. c) EEG part of the ICN-FG component. Note that as we stacked up all frequency-specific EEG connectomes for each subject (cf. Figure 2), we obtained a single EEG component part (c) associated with an ICA mixing weight for each subject and frequency (represented by one bar each in (a)). d) through f) visualize equivalent aspects for the VIS-FS component. All panels represent the generalization dataset (for main data see Figure 4. Colorbars have been saturated at 95th and 5th percentile for better comparison with Figures SI4 and SI5). VIS: Visual, SM: Somatomotor, DA: Dorsal Attention, VA: Ventral Attention, L: Limbic, FP: Fronto Parietal, DMN: Default Mode Network

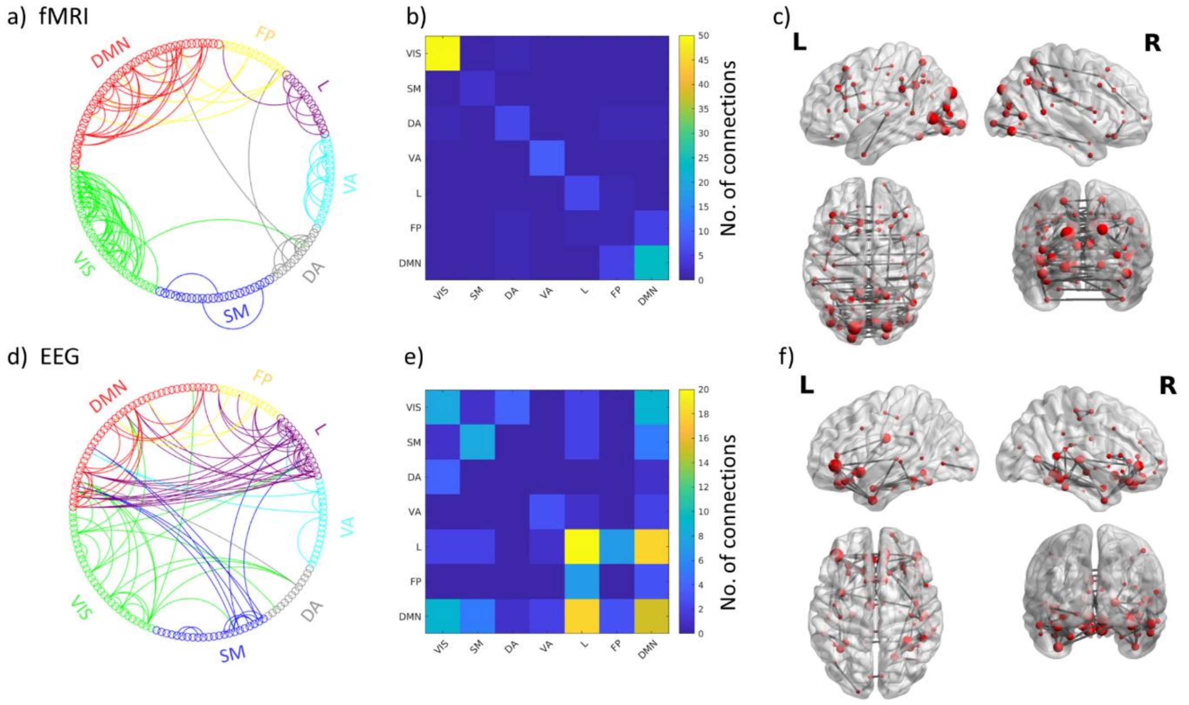

Figure SI4: Connections with highest nodal strength (99th percentile) for the ICN-FG component: Circle graphs show all connections between different ICN networks for fMRI (a) and EEG (d); Matrices summarize the number of connections

falling into each ICN-ICN pair for fMRI (b) and EEG (e); Brain renderings show strongest connections of the components on a canonical reconstructed cortical surface for the fMRI (c) and EEG (f) part of the hybrid component. Data correspond to the generalization dataset; for the results of the main dataset see Figure 5. VIS: Visual, SM: Somatomotor, DA: Dorsal Attention, VA: Ventral Attention, L: Limbic, FP: Fronto Parietal, DMN: Default Mode Network.

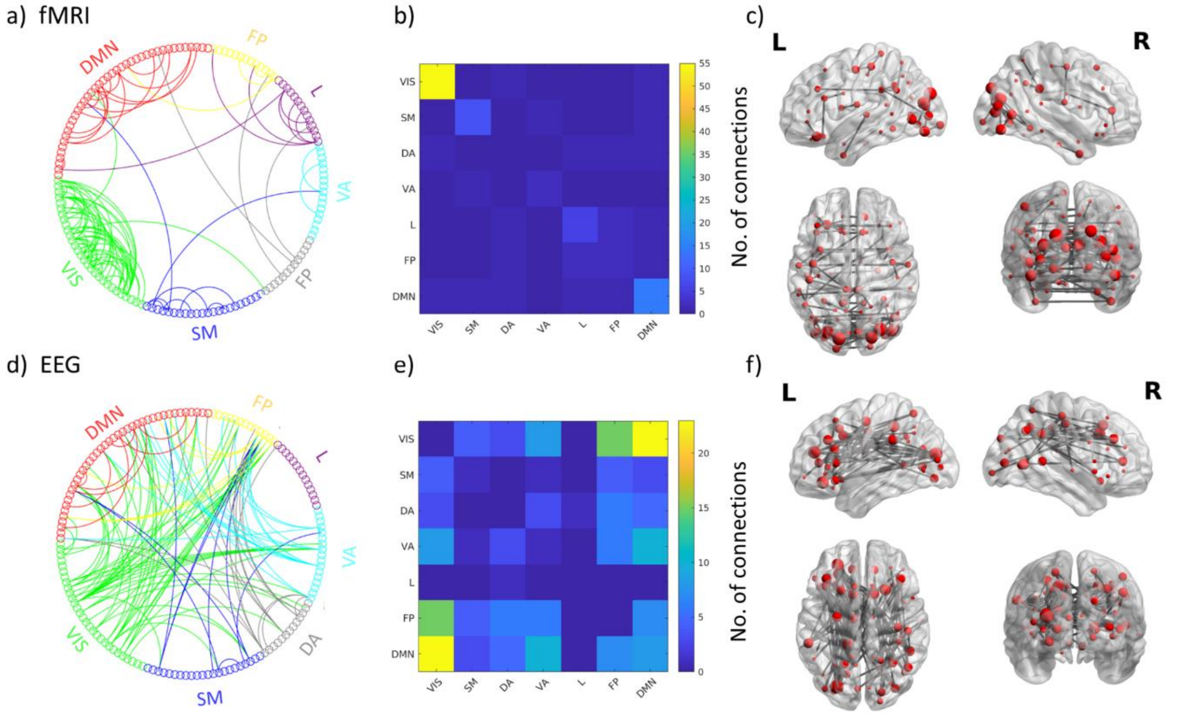

Figure SI5: Connections with highest nodal strength (99th percentile) for the VIS-FS component: Circle graphs show all connections between different ICN networks for fMRI (a) and EEG (d); Matrices summarize the number of connections falling into each ICN-ICN pair for fMRI (b) and EEG (e); Brain renderings show strongest connections of the components on a canonical reconstructed cortical surface for the fMRI (c) and EEG (f) part of the hybrid component. Data correspond to the generalization dataset; for the results of the main dataset see Figure 6. VIS: Visual, SM: Somatomotor, DA: Dorsal Attention, VA: Ventral Attention, L: Limbic, FP: Fronto Parietal, DMN: Default Mode Network.

	$ICC_{Frequency}$	$ICC_{Subjects}$	Q_{fMRI}	Q_{EEG}
Main Dataset ICN-FG				
IC5-PCA75	-0.03	0.83	0.15	0.07
IC5-PCA80	-0.0004	0.87	0.09	0.04
IC5-PCA85	-0.0004	0.86	0.17	0.04
IC5-PCA90	-0.02	0.82	0.09	0.02
IC10-PCA75	-0.008	0.81	0.21	0.09
IC10-PCA80	-0.0005	0.81	0.23	0.09
IC10-PCA85	0.009	0.85	0.15	0.03
IC10-PCA90	-0.03	0.84	0.06	0.02
IC15-PCA80	-0.02	0.92	0.21	0.06
IC15-PCA85	-0.01	0.91	0.15	0.07
IC15-PCA90	-0.02	0.87	0.07	0.02
IC20-PCA85	-0.006	0.91	0.15	0.07
IC20-PCA90	-0.01	0.91	0.12	0.05
IC20-PCA90	-0.01	0.91	0.12	0.07

Main Dataset VIS-FS				
IC5-PCA75	0.68	-0.11	0.22	-0.01
IC10-PCA75	0.69	-0.11	0.32	-0.01
IC10-PCA80	0.69	-0.11	0.26	-0.01
IC15-PCA85	0.69	-0.12	0.25	-0.01
IC20-PCA85	0.69	-0.12	0.29	-0.01
Generalization Dataset ICN-FG				
IC5-PCA75	0.32	0.41	0.38	0.15
IC10-PCA75	0.27	0.48	0.37	0.14
IC10-PCA80	0.20	0.43	0.37	0.15
IC15-PCA80	0.16	0.50	0.36	0.14
IC15-PCA85	0.36	0.12	0.37	0.12
IC20-PCA85	0.25	0.37	0.37	0.12
IC20-PCA90	0.22	0.38	0.37	0.13
Generalization Dataset VIS-FS				
IC5-PCA75	0.49	0.03	0.35	-0.02
IC5-PCA80	0.49	0.01	0.39	-0.01
IC10-PCA75	0.48	0.07	0.30	-0.02
IC10-PCA80	0.47	0.06	0.31	-0.02
IC10-PCA85	0.50	0.01	0.39	-0.01
IC15-PCA80	0.46	0.08	0.28	-0.02
IC15-PCA85	0.46	0.08	0.30	-0.02
IC20-PCA85	0.46	0.09	0.27	-0.02
IC20-PCA90	0.50	0.02	0.37	-0.01

SI Table 1:Evaluation of the complete explored parameter space (e.g. IC5PCA75 depicts an analysis using 5 ICs and keeping all PCs explaining 75% of the data variance.) and the corresponding ICC (using either the EEG frequency band weighting or the subject weighting of the IC mixing matrix as raters) and modularity (Q) values (of the EEG and fMRI component of the respective IC). Measurements are robust across the parameter space in the cases where stable ICs could be identified.

SI Results

Supplementary analysis of head motion in the main dataset

As a consequence of the observed correlation between the ICN-FG mixing weights and head motion in the main dataset, we extended the analysis of the main dataset to also look at a previously excluded stable component.

As described in the methods section, the ICN-FG component might split up into two similar components for the main dataset. Indeed, for the chosen parameter set (keeping PCs that explain 75% of the variance and calculating the ICA for 10 ICs), we found a second IC with similar properties to the ICN-FG component namely ICN-organization for EEG and fMRI part of the IC (q_{fMRI} = 0.11, q_{EEG}=0.06), subject specific fingerprint ($ICC_{subject}$=0.85, $p<10^{-10}$) and no differences of mixing weights as a function of EEG frequency band (ICC_{freq}=-0.018, p=0.71). A very small relationship between EEG and fMRI part of the IC weights was observed

(r=0.049116, p=2.9722 10^{-07}). The IC strengths were associated between the above-described second ICN-FG and the ICN-FG in the generalization dataset (fMRI-fMRI 0.25 p<1.0*10^{-300}; EEG-EEG = 0.42, p<1.0*10^{-300}). This correlation between main and generalization dataset was lower than the originally found ICN-FG component (both for EEG and fMRI).

Contrasting the observation for the originally found IC, the mixing weights of this IC were not related to movement (FD vs. mixing weights: rho=-0.12, p=0.18; No. of scrubbed volumes vs. mixing weights: rho=-0.02, p=0.78).
